# All HEPped Up about Methylation

**DOI:** 10.1371/journal.pbio.0020433

**Published:** 2004-11-23

For a recipe to become a meal, it's often necessary to embellish or modify the basic instructions—and to keep a note of the changes that work, so that it can be just as delicious next time around. The same is true for a gene, whose basic recipe—its nucleotide sequence—can be heritably annotated to “epigenetically” influence its level of expression without altering its sequence. Among the many epigenetic influences at work in the genome, methylation of cytosine is one of the most versatile and powerful. Addition of a methyl (-CH_3_) group to cytosines within a gene's regulatory regions can reduce its transcription. In its extreme form, methylation is involved in silencing one of the two X chromosomes in female mammals. Aberrant methylation underlies susceptibilities to several forms of cancer, and is likely to be involved in numerous other human diseases.

The goal of the Human Epigenome Project (HEP) is to map the methylation patterns of human genes, and to determine how they vary: among individuals, among tissues within an individual, and even over time within a single tissue. In this issue, Stephan Beck and colleagues describe the execution and results of a HEP pilot project, in which they analyzed methylation within the major histocompatibility complex (MHC), the set of genes that establish an individual's self-identity within the context of immune surveillance.

The key to any such large-scale project is high throughput—a rapid, efficient set of technologies that produce the needed data with minimal human intervention. The strategy used by Rakyan et al. included bisulfite sequencing of DNA, in which unmethylated cytosines are chemically converted to uracils, while methylated cytosines are not. Software they developed detects the methylated sites and provides an overall measure of the methylation level within any given sequence. They confirmed the accuracy of their method with mass spectrometry, an alternative method also suitable for high-throughput screening.

They initially analyzed 253 sequences within 90 genes in the MHC, about two-thirds of the total, from multiple tissues in multiple individuals. They found that most genes were either completely methylated or completely unmethylated, while relatively few had an intermediate value. The significance of this distribution pattern is not yet clear, although it does confirm similar results in smaller samples from other research groups. The researchers also confirmed that so-called CpG islands, regions rich in CG dinucleotides, are relatively hypomethylated, especially when they occur at the upstream end of a gene.[Fig pbio-0020433-g001]

**Figure pbio-0020433-g001:**
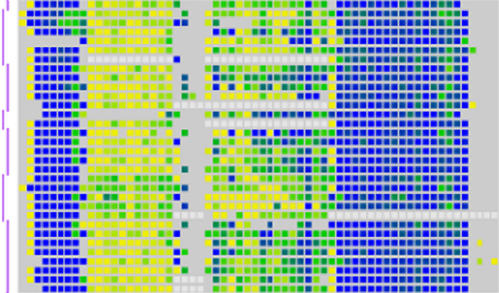
Methylation levels in a region of the human genome

Rakyan et al. also found differences in methylation levels among tissues, with some suggestion that the variations influence tissue-specific alternative splicing, at least in some genes. Intriguing inter-individual differences were also found, with median methylation levels differing significantly between individuals for at least one tissue at almost half the sites analyzed. For instance, such differences were found in liver for the regulatory region for the tumor necrosis factor gene.

A major goal of the HEP is to identify methylation variable positions, sites whose methylation state is linked with some important biological state, be it tissue type, developmental stage, or disease state. The pilot project described here begins this undertaking, which will be greatly expanded as the HEP progresses. The first phase of the full-scale HEP, an analysis of 5,000 DNA sequences, is currently underway.

